# Effects of overgrazing on the functional diversity of rodents in desert areas

**DOI:** 10.1002/ece3.10849

**Published:** 2024-02-21

**Authors:** Na Zhu, Xin Li, Xiaodong Wu, Linlin Li, Suwen Yang, Heping Fu, Shuai Yuan

**Affiliations:** ^1^ Key Laboratory of Grassland Rodent Ecology and Pest Controlled, Inner Mongolia Hohhot China; ^2^ Key Laboratory of Grassland Resources of the Ministry of Education Hohhot China; ^3^ College of Grassland, Resources and Environment Inner Mongolia Agricultural University Hohhot China; ^4^ College of Grassland Science Xinjiang Agricultural University Urumqi China

**Keywords:** functional diversity, functional traits, overgrazing, rodent community

## Abstract

Environmental stressors and disturbances can cause changes in an ecosystem's community structure, which can be reflected in its functional diversity. As grazing intensity increases, this causes changes in the environment that inevitably lead to changes in the community structure, which can especially affect rodents due to their sensitivity to the environment. The effects of grazing prohibition and overgrazing on the functional diversity of desert rodent communities in Alxa were studied in April, July, and October of 2018–2020. The trap‐day method was used to study rodent communities in disturbed habitats. Five functional traits were selected and quantified: nutrition, life history, physiology, morphology, and activity rhythm. The results showed that: (1) The species composition of rodent communities in the Alxa Desert in spring and autumn was significantly correlated with the functional traits of the hibernation, reproductive cycle, and feeding habits. The species composition in the summer was only significantly correlated with the functional traits of reproductive cycle and diet. (2) The effects of overgrazing on the functional diversity of rodents in desert areas have significant temporal and spatial characteristics. (3) In spring and summer, overgrazing made the Functional Richness index of the rodent community lower than that of areas where grazing is prohibited, but the Functional Evenness index was higher than that of grazing‐prohibition areas. In autumn, overgrazing increased the Functional Richness index of the rodent community and decreased the Functional Evenness index. The Functional Divergence index was higher in overgrazing areas than in grazing‐prohibited ones. These results suggest that, in spring and summer, overgrazing reduced the ecological space utilization ability of rodent communities; however, the impact on the degree of utilization of community resources is more comprehensive. In autumn, overgrazing increases the ability of rodent communities to use ecological space but reduces resource efficiency. Overgrazing makes the niche differentiation of rodent communities higher, the degree of overlap lower, and the competition between species weaker. Therefore, overgrazing will affect the functional diversity of the community through the utilization of ecological space, resource utilization, interspecific competition, and niche.

## Graphical abstract

In this study, the dynamic processes of rodent communities under different disturbances in desert areas were compared, and the effects of disturbance on the functional structure of rodent communities were revealed from the perspective of functional traits.

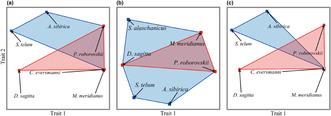

## 1 INTRODUCTION

In the past decade, functional diversity has been paid attention to and widely used. The field now recognizes it as an important form of biodiversity in addition to species diversity and genetic diversity (Aguirre‐Gutiérrez et al., 2017), and it has also become a research hotspot in community ecology (Jiang, 2010). Functional diversity refers to the functional traits and the range of their variation across all the species and organic matter in the community that affect the function of the ecosystem (Tilman, 2001). It can reflect the changes in community structure and the response to environmental stress (Petchey & Gaston, 2006, 2010) and play an important role in the dynamics, stability, productivity, nutrient balance, and other aspects of the ecosystem (Li et al., 2022; Odanaka & Rehan, 2019). Functional diversity can be used to quantify the species composition based on traits in ecosystems and the degree to which they influence ecosystems (Lin et al., 2022). Methods based on functional traits (Mouillot et al., 2013) can explain different aspects of the community from the various functional strategies of the species (Kirwan et al., 2009). Functional traits are any traits that directly affect the performance of organisms (Mouillot et al., 2013). In the long process of adapting to the environment, species may evolve any form (Myers et al., 2021), behavior (Sassi et al., 2015), or physiological characteristic (Lin, 1998; Riquelme et al., 2014) that is compatible with the environment. Differences in functional traits can not only objectively reflect different physiological processes and adaptations to the external environment, but they can also be sensitive to environmental changes and have many potential indicators of changes in communities and populations along environmental gradients (Tilman et al., 1997).

More and more evidence shows that species filtering caused by interference and competitive interactions is at least partially driven by the species' functional traits (Kreuzinger et al., 2022; Sfair et al., 2022; Walker et al., 2021). When a disturbance excludes species with specific traits or severely reduces their abundance, trait differences can drive interspecific differences in species' responses to that disturbance, including human disturbance, biological stress, and environmental change (Li et al., 2022; Mouillot et al., 2013). Therefore, research on functional traits has become the key to understanding interference responses to environmental stressors (Kirwan et al., 2009). Among all environmental disturbances, human disturbance has some of the most lasting effects on community diversity and traits. Grazing is one of the main forms of anthropogenic disturbance to natural grasslands and is a main driver of grassland community biodiversity, function, and stability (Liang et al., 2021). Moderate grazing has a positive effect on grassland ecosystems, as it can not only stabilize the grassland community but also enhance its anti‐interference abilities (Souther et al., 2020). Overgrazing significantly reduces species abundance (Archer et al., 2022; Yao et al., 2021) and causes soil exposure and fertility declines (Yang & Yang, 2022), grassland degradation (Wang et al., 2022), and habitat changes that indirectly change species diversity (Matteo et al., 2011; Stuart, 2019). Small mammals are key components of grassland ecosystems and are important indicators of habitat stability, biodiversity, and land use change (Schirmel et al., 2016). Grazing affects small mammals' spatial utilization, changes the abundance of their food resources (Jchac et al., 2022), increases their predation risk, and changes their life history strategies. It can also change their microhabitat environments, distribution (Anthony, 2008), body weight (Santini et al., 2017), feeding habits, field, feeding, and excavation behavior, and reproduction and life history characteristics (Su et al., 2016). Although the importance of functional diversity is being recognized by more and more researchers, there are still few theoretical and practical studies on the functional diversity of rodent communities.

In northern China, grasslands extend from the northeast to the west, and the climate becomes drier along this gradient. Located in Inner Mongolia, the Alxa is a typical temperate desert. Grazing is the main form of human disturbance in this area. Due to a long history of overgrazing, the ecosystem has become more fragile. Therefore, to understand the functional diversity of rodent communities in the Alxa desert, we propose the following hypotheses: Under overgrazing and grazing prohibition conditions, the functional diversity of rodent communities is different and has seasonal changes. Through the change in the functional diversity index, it shows the influence on the ecological space utilization, resource utilization, interspecific competition, and niche of the community.

## 2 METHODS

### 2.1 Study area

**FIGURE 1:**
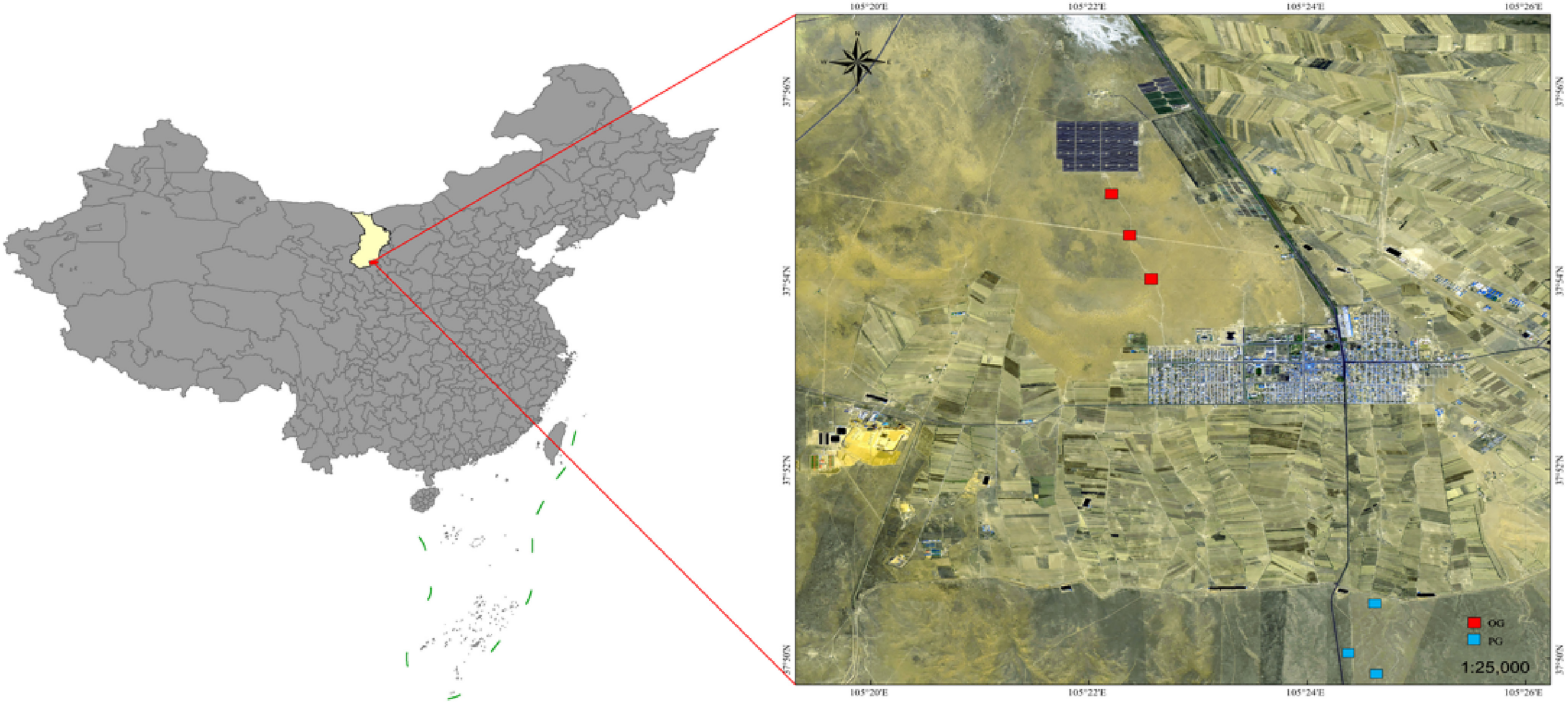
Map of study area. GP, grazing prohibition area; OG, overgrazing area.

The study area is located in the south of Alxa Left Banner (an administrative division of the Inner Mongolia Autonomous Region in China), at the eastern edge of the Tengger Desert (average altitude 800–1500 m, latitude 37°50′ N–37°56′ N, longitude 105°20′ E–105°26′ E) (Figure 1). The habitat is mainly desert and semi‐desert grassland, belonging to a temperate desert‐arid area. It has a typical continental climate, characterized by high winds and sand, drought and little rainfall, plentiful sunshine, and high evaporation. The annual rainfall is 80–220 mm, and the annual evaporation is 2900–3300 mm. Each year, there are an average of 3316 h of sunshine, an annual average temperature of 7.2°C, and a frost‐free period of 120–180 days. The soil is light brown calcareous soil, and the plants are mainly xerophytic, hyper xeric, halophytic, and sandy desert plants. There are a few perennial grasses and legumes. The dominant plant families are Chenopodiaceae, Compositae, and Zygophyllaceae, followed by Rosaceae and Tamaricaceae (Yuan et al., 2011).

### 2.2 Functional traits

Using the trap‐day method, three grazing‐prohibited areas and three overgrazing areas were randomly selected from the field experimental plots of the desert ecology and rodent control base in Luanjingtan of Alxa Left Banner from 2018 to 2020. In our study area, the overgrazing area was grazed continuously throughout the year, while the grazing‐prohibited area has been fenced since 1997. In the overgrazing sites, sheep grazing intensity was close to the prevailing grazing intensity in the local areas and was controlled within the range of 3.75–4.23 sheep per ha (Yang et al., 2020). Several seasons were sampled, with spring samples taking place in April, summer ones in July, and autumn ones in October. Three trap plots were randomly set up in the grazing‐prohibition area and overgrazing area in three seasons, and a total of six trap plots were set up in each season for four consecutive days. Each plot area was 10 hm^2^. Three trap lines were set up in each sample area, with 50 m between each trap line, and 100 traps per trap line. A total of 300 traps were placed in each plot, 1800 traps were placed in each season, and 16,200 traps were placed in 3 years. The capture rate in the overgrazing area was: the spring: 0.0838, the summer: 0.1111, and the autumn: 0.0838. The capture rate in grazing‐prohibition area was: the spring: 0.0561, the summer: 0.0383, and the autumn: 0.0144. The mouse trap was a standard medium iron plate trap, and fresh peanut rice was used as bait, and the trap was placed at 6 p.m. every day, and the trap was collected at 6 a.m. the next day as a trap day. The species, sex, body weight, body length, ear length, tail length, hind foot length, stomach content, and reproductive status of the captured individuals were measured and recorded. In this study, five functional traits related to the ecological function of rodents were selected (Keller et al., 2023; Li et al., 2022; Tsianou et al., 2021), as detailed in Table 1.

**TABLE 1:** Classification standard and types of functional traits.

Classification of function	Functional trait	Type of functional traits	Ecological function
Nutrition	Feeding habit	Phytophagous, omnivorous	Reflects the nutritional balance ability
Life history	Reproductive cycle	1/year, 2/year	The ability to reproduce
Physiology	Hibernation	Hibernating, nonhibernating	Reflects the ability to resist risks
Morphology	Limb morphology	Bipedal, quadrupedal	Reflects the style of locomotion
Activity rhythm	Activity rhythm	Diurnal, nocturnal	Reflects the usage of time

### 2.3 Species diversity

#### 2.3.1 Species accumulation curve

The species accumulation curve (SAC) can be used to judge the effectiveness of sampling, and its model describes the relationship between the increase in sampling amount and the change in species number, which is an effective tool to judge the adequacy of investigation (Ugland et al., 2003). If the curve always maintains an upward trend, it indicates that the sample size is insufficient. On the contrary, when the trend at the end of the curve tends to be gentle, it indicates that the sample size is sufficient.

#### 2.3.2 Chao1 index

The Chao1 index is used to reflect the index of species richness. The chao1 index was used to evaluate the number of species in a sample. The larger the chao1 index, the richer the community species. The calculation formula is as follows:

$$S_{\text{chao}1}=S_{\mathrm{obs}}+\frac{F_{1}\left(F_{1}-1\right)}{2\left(F_{2}+1\right)}$$

In the formula, *S*_obs_ is the number of species in the community, *F*_1_ is the species with an abundance of 1, and *F*_2_ is the species with an abundance of 2.

### 2.4 Kruskal‐Wallis test

The Kruskal‐Wallis test (*H* test) is used to infer whether there is a difference in the distribution of multiple populations from which multiple independent samples of a measurement sample or a grade sample are derived. The calculation process is as follows: (1) Mix the data of each group from small to large rank, and take the average rank when the data are equal; (2) set the number of cases in each group as $n_{i}\left(\sum n_{i}=N\right)$, the rank sum is *R*_*i*_, and the *H* value is calculated according to the following formula.

$$H=\frac{12}{N\left(N+1\right)}\left(\sum \frac{R_{i}^{2}}{n_{i}}\right)-3\left(N+1\right)$$

### 2.5 Beta diversity index

Using the Jaccard similarity coefficient (Si et al., 2017), the *β* diversity of rodent communities in the grazing‐prohibited and overgrazing areas of the Alxa desert was calculated. The calculation formula is as follows:

$$\beta_{\mathrm{JAC}}=\frac{b+c}{a+b+c}$$

In this formula, *a* is the number of species shared by the two communities (grazing‐prohibition and overgrazing); *b* is the number of species present in the first community and absent in the second community; *c* is the number of species present in the second community but not in the first community. The proportion of species spatial turnover components and nested components was used to indicate which components determine the *β* diversity of desert rodent communities in Alxa,

$$\beta_{\text{ratio}}=\frac{\beta_{\mathrm{JNE}}}{\beta_{\mathrm{JAC}}}$$

When *β*_ratio_ < 0.5, it indicates that Community *β* diversity is mainly determined by species spatial turnover (*β*_TUR_). When *β*_ratio_ > 0.5, it means that Community *β* diversity is mainly determined by nested components (*β*_NES_). *β*_JAC_ is the ratio of nested components to total diversity, *β*_JAC_ is the similarity index, and *β*_JNE_ is the nested component of the similarity index. *β*_ratio_ is the ratio of nested components to total *β* diversity; *β*_JAC_ is the similarity index; and *β*_JNE_ is the nested component of the similarity index.

### 2.6 Functional diversity

Functional diversity is measured by using functional traits to calculate a functional diversity index. The following are several functional diversity indexes that are widely used and have predictive effects on interference (Jiang, 2010; Mouillot et al., 2013; Zhang & Fan, 2011):

#### 2.6.1 Functional Richness (FR_*ic*_ )

This reflects the size of the functional space occupied by the organism in the community. The higher the index, the higher the ability to use ecological space. The calculation method is as follows: first, find out the species with extreme trait values and use them as the endpoints of the minimum convex polygon in the n‐dimensional trait space; then, connect the endpoints into the minimum convex polygon; and finally, calculate the area or volume of the minimum convex polygon. The number of species is required to be greater than the number of eigenvalues. The calculation formula is as follows:

$${\mathrm{FR}}_{\mathrm{ic}}=\frac{{\mathrm{SF}}_{\mathrm{ci}}}{R_{c}}$$

In the formula, FR_*ic*_ is the Functional Richness of trait *c* in community *i*, SF_*ci*_ is the niche occupied by species in the community, and *R*_*c*_ is the absolute value range of trait *c*.

#### 2.6.2 Functional Evenness (FE_ve_ )

This reflects the uniformity of the distribution of functional traits of organisms in the community in ecological space and the degree to which species in the community utilize effective resources. A higher index indicates more comprehensive utilization of effective resources and higher efficiency. First, the distance between all paired species is calculated, and then the minimum spanning tree in the multidimensional trait space is obtained by clustering according to the weight of each species abundance. Finally, the uniformity of the branch length of the minimum‐spanning tree is measured. The calculation formula is as follows:

$$\text{dist}\left(i,j\right)=\sqrt{{\left(a_{i}-a_{j}\right)}^{2}+{\left(b_{i}-b_{j}\right)}^{2}+\cdots +{\left(m_{i}-m_{j}\right)}^{2}}$$

$${\mathrm{EW}}_{l}=\text{dist}\left(i,j\right)/\left(w_{i}+w_{j}\right)$$

$${\mathrm{PEW}}_{l}={\mathrm{EW}}_{l}/\sum_{l=1}^{S-1}{\mathrm{EW}}_{l}$$

$${\mathrm{FE}}_{\mathrm{ve}}=\frac{\sum_{l=1}^{S-1}\min\left({\mathrm{PEW}}_{l}-\frac{1}{S-1}\right)-\frac{1}{S-1}}{1-\frac{1}{S-1}}$$

In the formula, *a*–*m* represents the five functional traits of species *i* and *j* in the functional space, dist (*i*, *j*) represents the Euclidean distance between species *i* and *j*, EW_*l*_ is the branch length, *w*_*i*_ and *w*_*j*_ are the relative abundance of species *i* and *j*, respectively, PEW_*l*_ is the weight of branch length, and *S* is the number of species.

#### 2.6.3 Functional Divergence (FD_iv_ )

This can reflect the degree of niche complementarity between species. A higher index indicates stronger niche complementarity between species and weaker competition. The dominant species are distributed at the edge of the functional space, which reflects a higher degree of niche differentiation and weaker resource competition among species. On the other hand, if the dominant species is near the center of the functional space, there will be a smaller index and fierce competition between species (Wang et al., 2021). The calculation formula is as follows:

$$g_{k}=\frac{1}{S}\sum_{i=1}^{S}x_{\mathrm{ik}}$$

$${\mathrm{dG}}_{i}=\sqrt{\sum_{k=1}^{T}{\left(x_{\mathrm{ik}}-g_{k}\right)}^{2}}$$

$$\bar{\mathrm{dG}}=\frac{1}{S}\sum_{i=1}^{S}{\mathrm{dG}}_{i}$$

$$∆d=\sum_{i=1}^{S}w_{i}\times \left({\mathrm{dG}}_{i}-\bar{\mathrm{dG}}\right)$$

$$∆\left|d\right|=\sum_{i=1}^{S}w_{i}\times \left|{\mathrm{dG}}_{i}-\bar{\mathrm{dG}}\right|$$

$${\mathrm{FD}}_{\mathrm{iv}}=\frac{∆d+\bar{\mathrm{dG}}}{∆\left|d\right|+\bar{\mathrm{dG}}}$$

In the formula, *S* is the number of species, *X*_*ik*_ is the value of trait *k* of species *i*, *g*_*k*_ is the center of gravity of trait *k*, *T* is the number of traits, *dG* is the average distance between species *i* and the center of gravity, Δ*d* is the dispersion degree weighted by abundance, and *w*_*i*_ is the relative abundance of species *i*.

### 2.7 Statistical analysis

Microsoft Excel was used to input and sort the original data and calculate the species diversity. A one‐way analysis of variance (ANOVA) was performed using SPSS 20.0 software and plotted using OriginPro 8 software. The Species Accumulation Curve was calculated and plotted using the “vegan” package of software R 4.2.0. The “mFD” package of software R 4.2.0 was used to analyze the correlation between the functional axis and species traits, *β* diversity, Jaccard similarity coefficient, Kruskal‐Wallis test, and functional diversity index calculation, and the “ggplot2” package was used for mapping.

## 3 RESULTS

### 3.1 Rodent community composition

#### 3.1.1 Species diversity

**FIGURE 2:**
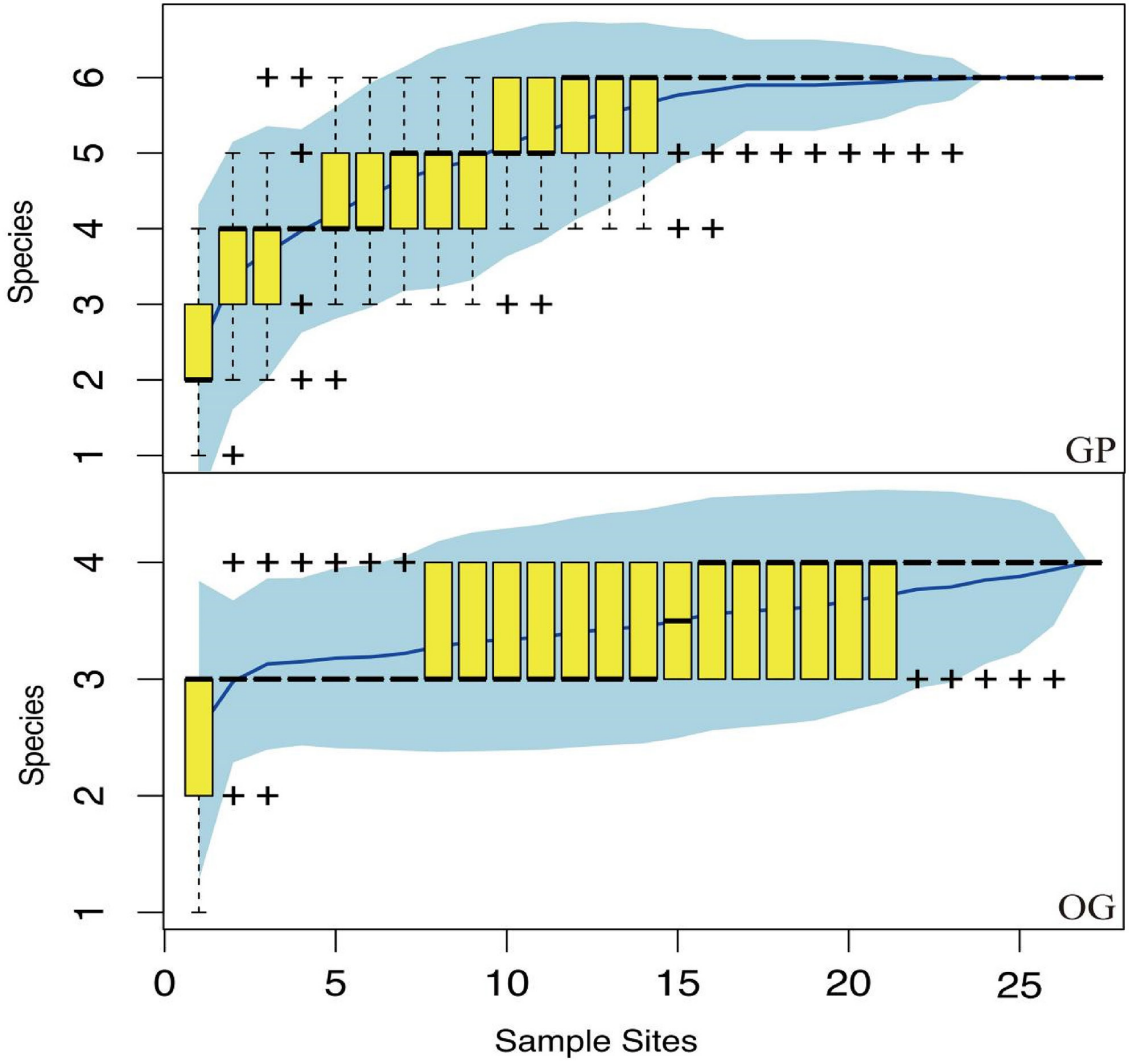
Species accumulation curve. GP, grazing prohibition area; OG, overgrazing area.

During the experiment, a total of 544 rodents from three families were captured in the grazing‐prohibited and overgrazing areas, including the midday gerbil (*Meriones meridianus*), the Roborovski hamster (*Phodopus roborovskii*), and the Eversmann's hamster (*Allocricetulus eversmanni*). There were three species of Dipodidae, including the Mongolian five‐toed jerboa (*Orientallactaga sibirica*), the northern three‐toed jerboa (*Dipus sagitta*), and the Mongolian jerboa (*Stylodipus andrewsi*). There was a single species of Sciuridae, namely the Alxa ground squirrel (*Spermophilus alaschanicus*). In the two communities of grazing prohibition and overgrazing, the upward trend of the species accumulation curve based on sampling times was stable and then gradually became an asymptote, indicating that the sampling amount of the two communities was sufficient (Figure 2) and the species richness of the community in the grazing prohibition area (Table 2).

**TABLE 2:** Chao1 index.

	GP1	GP2	GP3	OG1	OG2	OG3
Chao1 index	5	4	6	3	4	3

#### 3.1.2 Functional composition of community

**FIGURE 3:**
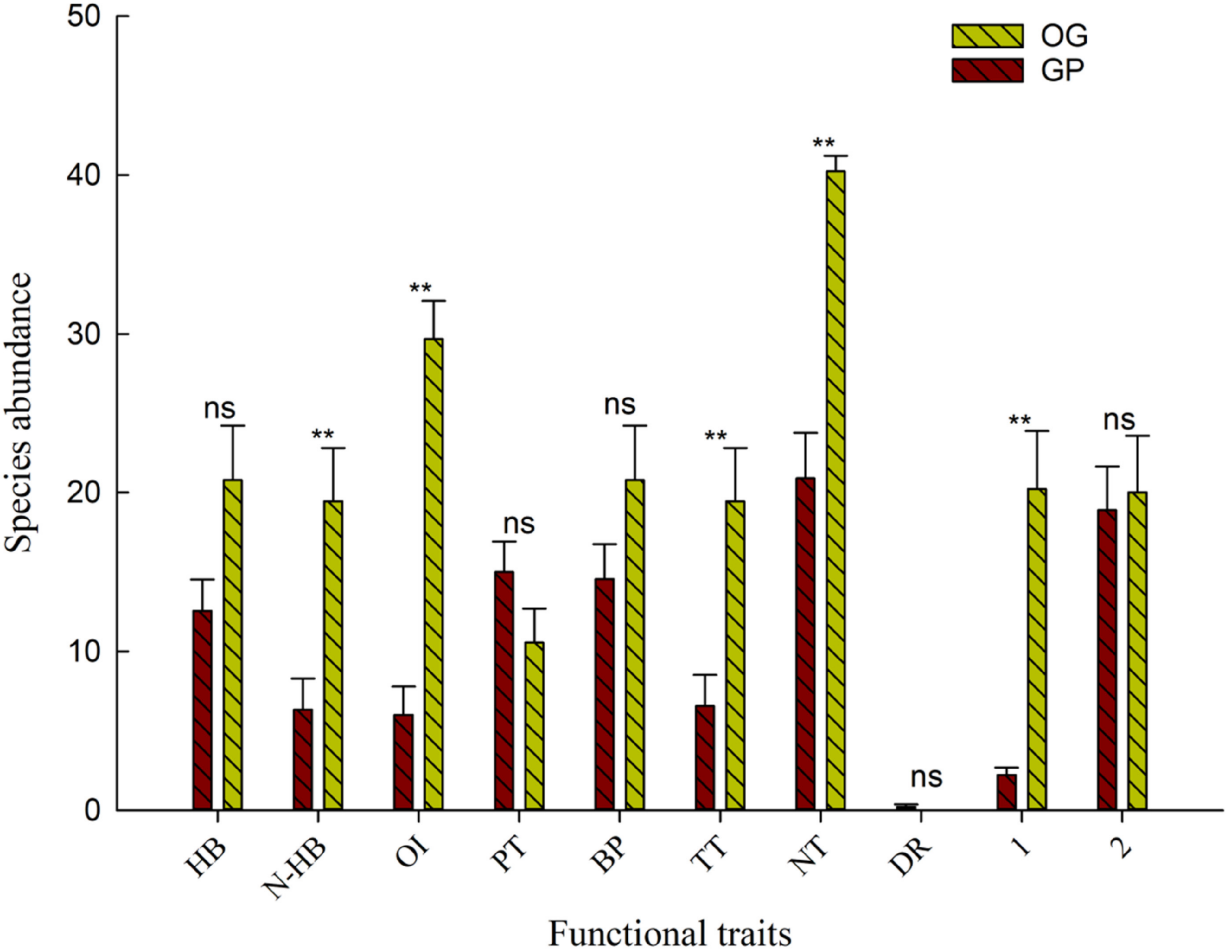
Differences in functional traits between different grazing patterns. GP, grazing‐prohibition; OG, overgrazing; ns means not significant (*p* > .05), **means very significant (*p* < .01).

Species composition and abundance varied between the different grazing habitats (Table 3). The population densities of species that had the functional traits of non‐hibernation, omnivory, quadrupedalism, nocturnality, and having one generation/year were significantly different between the grazing‐prohibited and overgrazing areas (*F*_non‐hibernation_ = 11.311, *p* = .004; *F*_omnivorous_ = 62.235, *p* = .001; *F*_tetrapods_ = 10.929, *p* = .004; *F*_nocturnal=_40.261, *p* = .001; *F*_generation/year_ = 23.729, *p* = .001). On the other hand, the population densities of species that had the functional traits of hibernation, phytophagy, bipedalism, multiple generations/year, and diurnality were not significantly different between overgrazing and grazing‐prohibition areas (*F*_hibernation_ = 4.271, *p* = .055; *F*_phytophagous_ = 2.402, *p* = .141; *F*_bipedal_ = 2.316, *p* = .148; *F*_diurnal_ = 2.286, *p* = .15; *F*_multigeneration/year_ = 0.061, *p* = .809) (Figure 3).

**TABLE 3:** Community composition.

Functional traits	Species		Number of individuals	
	Grazing‐prohibition	Overgrazing	Grazing‐prohibition	Overgrazing
HB	*Orientallactaga sibirica*, *Stylodipus andrewsi*, *Dipus sagitta*, *Spermophilus alaschanicus*	*D. sagitta*	113	187
N‐HB	*Meriones meridianus*, *Phodopus roborovskii*, *Cricetulus eversmanni*	*M. meridianus*, *P. roborovskii*, *C. eversmanni*	57	175
OI	*M. meridianus*, *D. sagitta*, *C. eversmanni*, *S. alaschanicus*	*M. meridianus*, *D. sagitta*, *C. eversmanni*	54	267
PT	*O. sibirica*, *S. andrewsi*, *P. roborovskii*	*P. roborovskii*	135	95
QP	*M. meridianus*, *P. roborovskii*, *S. alaschanicus*, *C. eversmanni*	*M. meridianus*, *P. roborovskii*, *C. eversmanni*	59	175
BP	*O. sibirica*, *D. sagitta*, *S. andrewsi*	*D. sagitta*	111	187
NT	*M. meridianus*, *O. sibirica*, *D. sagitta*, *S. andrewsi*, *P. roborovskii*, *C. eversmanni*	*M. meridianus*, *C. eversmanni*, *P. roborovskii*, *D. sagitta*	188	362
DR	*S. alaschanicus*		2	0
1	*S. andrewsi*, *D. sagitta*, *S. alaschanicus*	*D. sagitta*	20	187
2	*M. meridianus*, *O. sibirica*, *C. eversmanni*, *P. roborovskii*	*M. meridianus*, *C. eversmanni*, *P. roborovskii*	170	180

*Note*: 1: Generation/year, 2: multigeneration/year.

Abbreviations: BP, bipedal; DR, diurnal; HB, hibernation; N‐HB, non‐hibernation; NT, nocturnal; OI, omnivorous; PT, phytophagous; QP, quadrupedal.

### 3.2 *β* diversity of rodent community

**FIGURE 4:**
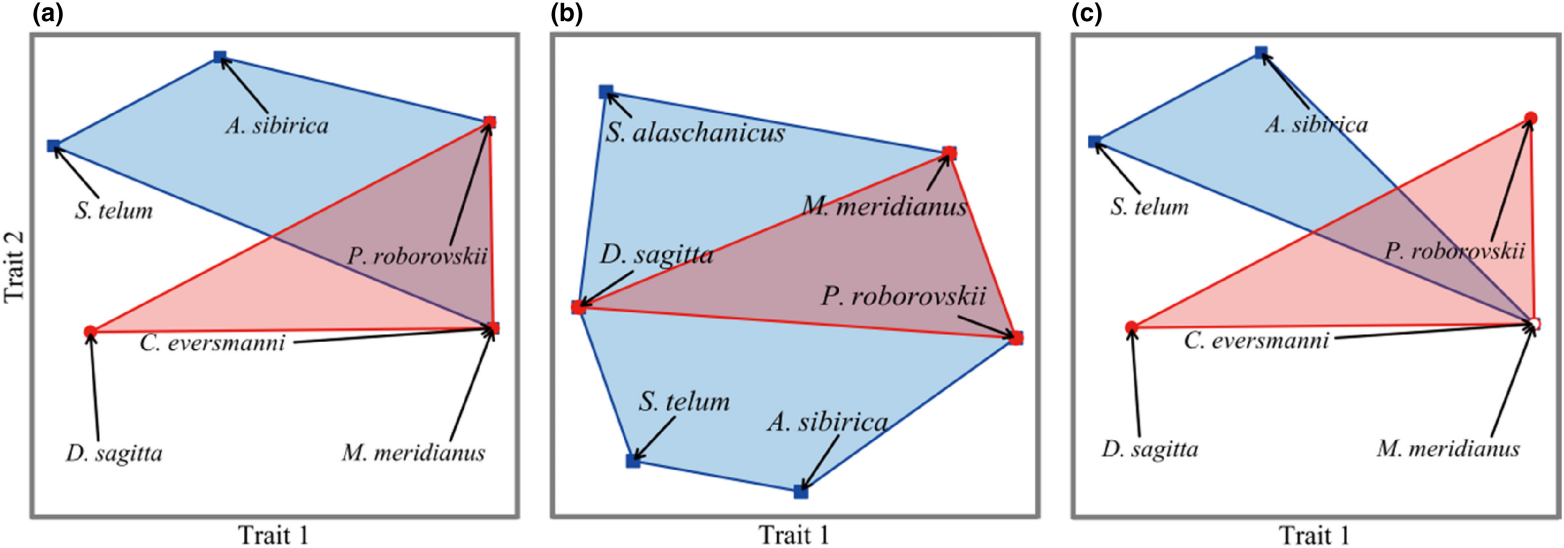
Beta diversity of the communities in different seasons. (a) Spring, (b) summer, and (c) autumn; the blue dots and polygons represent the species distribution points and ranges in the grazing‐prohibition areas; the red dots and polygons represent the distribution points and range of species in the overgrazing areas. *Meriones meridianus*: midday gerbil, *Phodopus roborovskii*: Roborovski hamster; *Allocricetulus eversmanni*: Eversmann's hamster; *Orientallactaga sibirica*: Mongolian five‐toed jerboa; *Dipus sagitta*: northern three‐toed jerboa; *Stylodipus andrewsi*: Mongolian jerboa; *Spermophilus alaschanicus*: Alxa ground squirrel.

In order to construct functional space, the correlation between functional axis and species traits was analyzed by the Kruskal‐Wallis test (*H* test), and the traits with significant correlation were used as abscissa and ordinate of functional space. The results showed that there were differences in the species distribution and functional composition of each community in different seasons. The community composition in spring and autumn was significantly correlated with functional traits such as hibernation (*p* < .05), reproductive cycle (*p* < .05), and feeding habit (*p* < .05). The community composition in summer was significantly correlated with functional traits such as reproductive cycle (*p* < .05) and diet (*p* < .05). These functional traits can be considered the main driving factors for the species composition of desert rodents in different seasons in the Alxa. Functional space is a multidimensional space with functional traits as the coordinate axes, and species are located in the position corresponding to their functional traits (Mouillot et al., 2013). Each axis corresponds to the original functional traits or to a composite of several original traits. Therefore, according to the above correlation results, it can be concluded that the functional traits significantly correlated with the first axis of the functional space (Trait 1) are hibernation and reproduction cycles, indicating that the first axis represents a composite of hibernation and reproduction cycles; the functional trait that was significantly correlated with the second axis (Trait 2) was feeding habits, indicating that the second axis represents diet, and the species were distributed in the corresponding positions. Jaccard similarity was used to explain community similarity. The *β* diversity changed differently over time (Figure 4). The spring *β* diversity (*β*_JAC_) was 0.7221, the spatial turnover component (*β*_TUR_) was 0.6254, and the nested component (*β*_NES_) was 0.0967. The *β* diversity in summer was 0.6883, the spatial turnover component was 0, and the nested component was 0.6883. The *β* diversity in autumn was 0.8704, the spatial turnover component was 0.8562, and the nested component was 0.0142. These results show that the Jaccard index was higher in the spring and autumn, indicating that the similarity between the two communities was high and the difference had no statistically significant differences. The *β* diversity of the rodent community was mainly determined by species spatial turnover, that is, *β*_ratio_ < 0.5. The Jaccard index in the summer was low, indicating significant differences between the two communities, including the species abundance and composition of the community, and the *β* diversity of the community was mainly determined by species nesting, that is, *β*_ratio_ > 0.5.

### 3.3 Functional diversity

**FIGURE 5:**
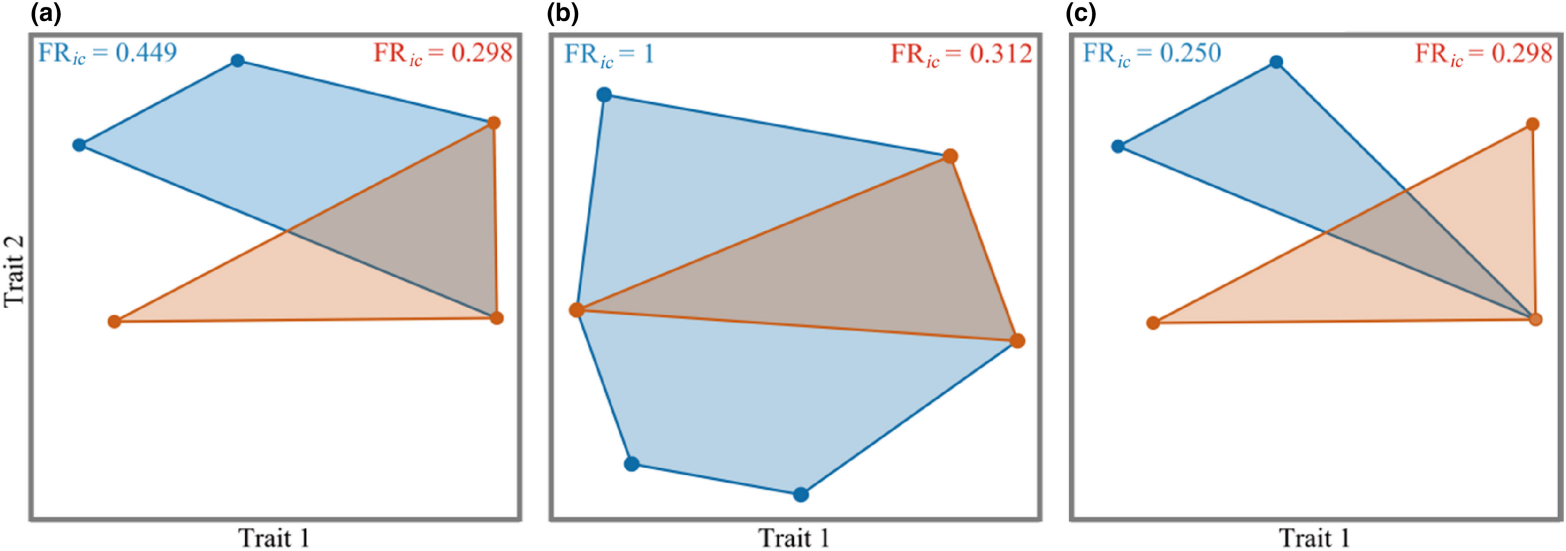
Functional Richness index (FR_*ic*_) of rodent communities in different seasons. (a) Spring, (b) summer, and (c) autumn; the blue dots and polygons represent the species distribution points and ranges of the grazing‐prohibition areas; the red dots and polygons represent the distribution points and range of species in the overgrazing areas.

Functional Richness reflects the utilization of community ecological space. The Functional Richness of overgrazing areas in the spring (FR_*ic*_ = 0.298) and summer (FR_*ic*_ = 0.312) was lower than that of grazing‐prohibition areas (Figure 5a,b), indicating that under conditions of overgrazing, there are fewer species, which reduces the Functional Richness and the utilization of community ecological space. The species in the grazing‐prohibition areas occupy more niches and have higher species diversity, which makes the ecological space utilization of the community higher. The ecological space utilization of the grazing‐prohibition and overgrazing communities is significantly different across seasons. The Functional Richness of the overgrazing areas (FR_*ic*_ = 0.298) in autumn is higher than that of the grazing‐prohibition areas (Figure 5c), indicating that due to the differences in the community composition in the overgrazing areas, the uniform distribution of non‐hibernation and hibernating species makes the ecological space utilization of the overgrazing areas higher. On the whole, overgrazing will increase the functional richness index of the community, which will affect the ecological space utilization of the rodent community, and there are seasonal differences.

**FIGURE 6:**
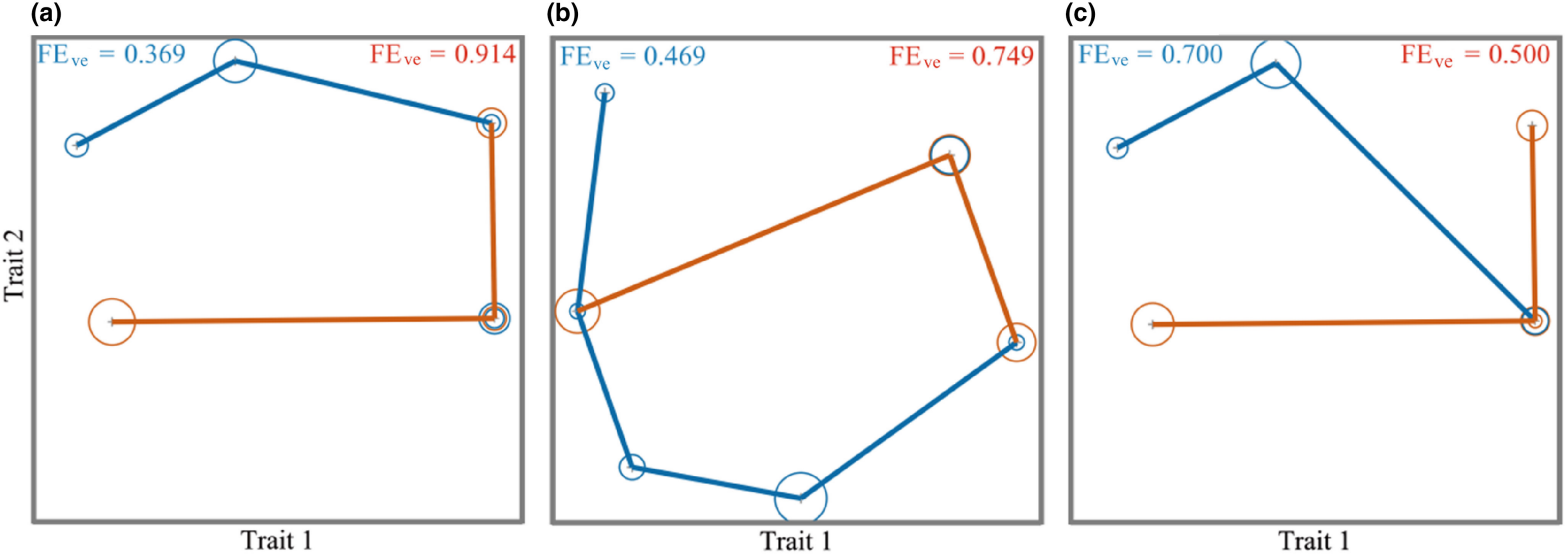
Functional Evenness index (FE_ve_) of rodent communities in different seasons. (a) Spring, (b) summer, and (c) autumn; the size of the circle in the figure represents the abundance of species in the community. A larger circle means more species, and a smaller circle means fewer species. The blue dots and lines represent the species and abundance of the grazing‐prohibition areas, and the red dots and lines represent the species and abundance of the grazed areas.

Functional Evenness reflects the degree of utilization of effective community resources. The Functional Evenness of overgrazing areas was significantly higher than that of grazing‐prohibition areas in the spring (FE_ve_ = 0.914) and summer (FE_ve_ = 0.749), (Figure 6a,b), indicating that the overgrazing communities differentiated niches due to disturbance and that the resources could be fully utilized. The Functional Evenness of overgrazing areas (FE_ve_ = 0.500) decreased in the autumn (Figure 6c), and that of grazing‐prohibition areas (FE_ve_ = 0.700) increased, indicating that the difference in food resources and community composition in overgrazing area led to a decrease in degree of resource utilization. On the whole, overgrazing will make the community function uniformity index higher, and the overgrazing community can make full use of the resources of the environment and form a more stable community.

**FIGURE 7:**
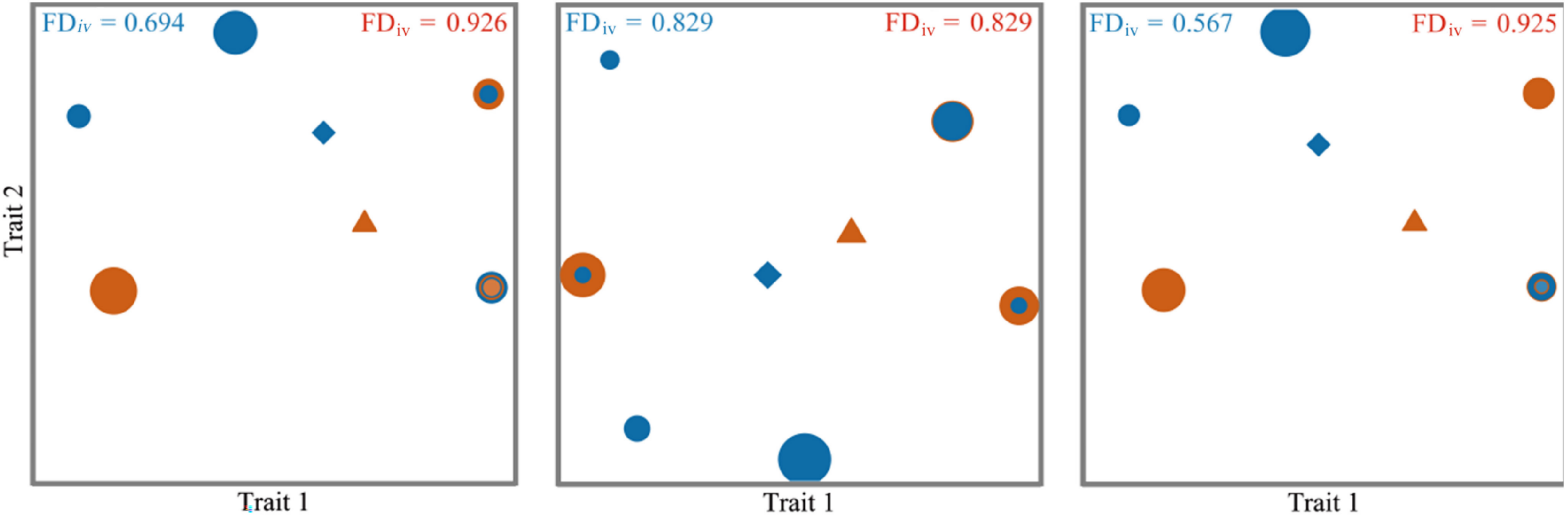
Functional Divergence index (FD_iv_) of rodent communities in different seasons. (a) Spring, (b) summer, and (c) autumn; the size of the solid circle in the figure represents the abundance of the species in the community, and the diamond and triangle represent the center of gravity of the community in the grazing‐prohibition areas and in the overgrazing areas. The blue circle represents the species and abundance in the grazing‐prohibition areas, and the red circle represents the species and abundance in the overgrazing areas.

Functional Divergence reflects the competition between community species. The Functional Divergence of overgrazing areas was significantly higher than that of grazing‐prohibition areas in the spring (FD_iv_ = 0.926) and autumn (FD_iv_ = 0.925) (Figure 7a,c), indicating disturbances tend to make the community functionally heterogeneous, resulting in high niche differentiation, stronger complementarity of resource utilization, and low resource competition between species in overgrazing areas. The Functional Divergence of the overgrazing areas and the grazing‐prohibition areas is the same (FD_iv_ = 0.829) in the summer (Figure 7b), indicating that the summer hydrothermal conditions are better and the food resources are more abundant, so there is no difference in species competition between communities. Overall, grazing increased the dispersion of community function, indicating that overgrazing affects communities by influencing competition between species.

## 4 DISCUSSION

### 4.1 Functional traits of rodents

The main driving factors of the rodent community composition in the Alxa desert in the spring and autumn were hibernation, reproductive cycle, and feeding habits. The main driving factors for the species composition in the summer are the reproductive cycle and feeding habits. In highly variable desert systems, niche filtering shows that species converge in the use of abundant resources, while diet as a divergent trait dominates on a smaller spatial scale (Rodríguez & Ojeda, 2014). Hibernation is an adaptive behavior that can help animals survive winter food shortages and reduce energy consumption during the winter. The communities in the study area are composed of both lipid‐storing hibernating species and non‐hibernating species, and their activities change seasonally. Before hibernation, rodents usually increase their food intake in the summer and autumn to store fat or nutrients (Zhu et al., 2021). In the spring, the body temperatures and metabolic rates of rodents gradually recover after hibernation, and they begin to move and feed (Mao et al., 2022). The reproductive cycle of the populations has seasonal dynamics, which are mainly determined by the characteristics of seasonal reproduction and the baseline population each season (Zhou et al., 2000). Generally, reproduction begins in the spring, soon enters the peak period, and continues into the summer. However, due to the low baseline population in the spring, the reproductive level is very low. After summer begins, due to the addition of the young born in spring and the continued reproduction of the summer population, the reproductive levels become higher and reach the peak of the reproductive period in 1 year. In the autumn, only some individuals reproduce in the population, so the reproductive levels become lower. In the winter, hibernating and nonhibernating populations have stopped reproductive, reaching the lowest levels of reproductive throughout the year. In the two communities of grazing‐prohibition and overgrazing areas, it was found that the Mongolian five‐toed jerboa (*Orientallactaga sibirica*) was an indicator species in the grazing‐prohibition area, and bipedality and phytophagy were indicator traits, whereas the Northern three‐toed jerboa (*D. sagitta*) was an indicator species in the overgrazing area, and bipedality and omnivory were indicator traits. In this way, the indicator traits are useful for future studies of rodent communities to detect environmental changes (Peng et al., 2022).

### 4.2 Functional diversity of rodent communities

The species composition and functional traits that differ between habitats will change after disturbance (Gorczynski et al., 2021). Disturbance alters the number and dominant species of rodents by altering their habitat conditions (Smith et al., 2022). Disturbances such as environmental stress and resource shortages can limit species diversity (Etard et al., 2022). With the occurrence of disturbances and changes in soil quality, the structure, functional composition, and functional diversity of small and medium‐sized animal communities change significantly. Human disturbance is not only the driving force behind functional diversity, but it also acts as a filter for functional diversity. Grazing disturbances, especially overgrazing, affect the food resources and niches of species by changing the plant biomass and community structure (Szabó et al., 2022). Due to the sensitivity of rodents to their environment, environmental changes inevitably lead to changes in their community structure. Combined with the filtering effect of the environment, species with functional traits adapted to the previous environment may become maladapted, and species that are not adapted to the environment may decline or even become extirpated (Rocabado et al., 2012). The functional space occupied by the community can be changed in different ways under different grazing regimes. The results showed that the functional diversity of the community differed greatly between grazing‐prohibited and overgrazing areas, with clear spatial and temporal differences.

The results show that the Functional Richness index of overgrazing areas in the spring and summer was lower than that of grazing‐prohibition areas, but it increased in the autumn. This index generally increased with an increase in species richness, indicating that the utilization rate of community ecological space increased and Functional Richness decreased with an increase in grazing intensity. Overgrazing had a greatly negative impact on the rodent community and could only allow species with suitable adaptations to survive (Tsianou et al., 2021). The grazing‐prohibition areas are always the best‐used habitats and can accommodate more species. Because of the availability of habitats and resources, more species with different functional traits can coexist. The evenness index of community function in the overgrazing areas was higher than that in grazing‐prohibition areas in the spring and summer, but it decreased in the autumn, indicating that the utilization of community resources in overgrazing areas was more comprehensive. One possible reason was that the spring community was preparing for reproduction and sharing resources. Overgrazing communities with omnivorous species would increase the utilization of resources and make rodent communities more stable (Chen et al., 2022). It may also be because the increase in food resources in the summer can support the coexistence of different trophic groups, which can provide more stable and longer‐term food resources and good living conditions for the community (Marcacci et al., 2022). The Functional Divergence Index was higher in the overgrazing areas than in the grazing prohibition areas, indicating that in each season, the dominant species in the grazing prohibition areas were close to the center of gravity of the functional space, the niche space overlap effect was strong, and the resource competition among species was fierce (Jiang, 2010; Zhang & Fan, 2011). However, overgrazing increased the functional heterogeneity of the community (He et al., 2021), so that the dominant species were at the edge of the functional space, the niche differentiation of the community was high, and the rodents used the existing resources in different ways (Zhang et al., 2019). In addition, due to differences in food resources between seasons, the overgrazing community has dominant species with a smaller body size (Yuan et al., 2013) in order to adapt to the harsh environment because the existence of rodents occupying smaller niches reduces the competition for food resources, can reduce the degree of niche overlap, and makes full use of food resources (Tinoco et al., 2018). This is also a reasonable explanation for the decline in the utilization rate of food resources in pastoral areas in the autumn. The above results are consistent with our hypothesis.

## 5 CONCLUSION

By analyzing the functional diversity of rodent communities, it was found that the functional diversity of rodent communities in grazing‐prohibited and overgrazing areas was different, and there were seasonal differences. The results supported our hypothesis that overgrazing would affect the functional diversity of the community by affecting the utilization of ecological space, resource utilization, interspecific competition, and niches. Distinguishing niches in disturbed communities based on functional traits and understanding how functional diversity changes along time scales, and spatial scales can help predict the potential response of rodent communities to disturbances. In future studies, it will be necessary to explore new functional traits and functional diversity indexes that can more fully capture the community ecological information, to more fully understand the community dynamics.

## AUTHOR CONTRIBUTIONS

**Na Zhu:** Formal analysis (equal); visualization (equal); writing – original draft (equal). **Xin Li:** Formal analysis (equal); visualization (equal). **Xiaodong Wu:** Data curation (equal); validation (equal). **Linlin Li:** Investigation (equal). **Suwen Yang:** Investigation (equal). **Heping Fu:** Validation (equal); writing – original draft (equal). **Shuai Yuan:** Data curation (equal); validation (equal).

## FUNDING INFORMATION

This experiment was funded by the National Natural Science Foundation of China (32060256, 32060395), Major Science and Technology Project of Inner Mongolia Autonomous Region (2021ZD0006), Inner Mongolia Nature Foundation (2023MS03025), The 2022 Inner Mongolia Autonomous Region Youth Science and Technology Talent Development Plan (NJYT22044), Science and Technology Project of Inner Mongolia Autonomous Region (2021GG0108), Basic scientific research business expenses of universities directly under Inner Mongolia Autonomous Region (BR22‐13‐07, BR220106, BR221037), Grassland Ecological Protection and Restoration Treatment Subsidy(RK2200000355).

## CONFLICT OF INTEREST STATEMENT

The authors declare that the research was conducted in the absence of any commercial or financial relationships that could be construed as a potential conflict of interest.

### OPEN RESEARCH BADGES

This article has earned an Open Data badge for making publicly available the digitally‐shareable data necessary to reproduce the reported results. The data is available at [https://github.com/istaude/neophytes‐hybrids].

## Data Availability

Data supporting the results of this study have been uploaded on the Dryad website. The data will be stored in https://datadryad.org/stash/share/Xw88Pxf‐2aVyFxHqA5w7PeDn50V54au8CvkegxpcoPQ.
